# Anabolic androgenic steroids exert a selective remodeling of the plasma lipidome that mirrors the decrease of the de novo lipogenesis in the liver

**DOI:** 10.1007/s11306-019-1632-0

**Published:** 2020-01-10

**Authors:** David Balgoma, Sofia Zelleroth, Alfhild Grönbladh, Mathias Hallberg, Curt Pettersson, Mikael Hedeland

**Affiliations:** 1 Analytical Pharmaceutical Chemistry, Department of Medicinal Chemistry, Uppsala University, Uppsala, Sweden; 2 The Beijer Laboratory, Biological Research on Drug Dependence, Department of Pharmaceutical Biosciences, Uppsala University, Uppsala, Sweden; 3 Uppsala Biomedicinska Centrum BMC, Husargatan 3, Box 574, 751 23 Uppsala, Sweden

**Keywords:** Androgens, Androgen receptor, Estrogen receptors, Lipidomics, Liver X receptors

## Abstract

**Introduction:**

The abuse of anabolic androgenic steroids (AASs) is a source of public concern because of their adverse effects. Supratherapeutic doses of AASs are known to be hepatotoxic and regulate the lipoproteins in plasma by modifying the metabolism of lipids in the liver, which is associated with metabolic diseases. However, the effect of AASs on the profile of lipids in plasma is unknown.

**Objectives:**

To describe the changes in the plasma lipidome exerted by AASs and to discuss these changes in the light of previous research about AASs and de novo lipogenesis in the liver.

**Methods:**

We treated male *Wistar* rats with supratherapeutic doses of nandrolone decanoate and testosterone undecanoate. Subsequently, we isolated the blood plasma and performed lipidomics analysis by liquid chromatography-high resolution mass spectrometry.

**Results:**

Lipid profiling revealed a decrease of sphingolipids and glycerolipids with palmitic, palmitoleic, stearic, and oleic acids. In addition, lipid profiling revealed an increase in free fatty acids and glycerophospholipids with odd-numbered chain fatty acids and/or arachidonic acid.

**Conclusion:**

The lipid profile presented herein reports the imprint of AASs on the plasma lipidome, which mirrors the downregulation of de novo lipogenesis in the liver. In a broader perspective, this profile will help to understand the influence of androgens on the lipid metabolism in future studies of diseases with dysregulated lipogenesis (e.g. type 2 diabetes, fatty liver disease, and hepatocellular carcinoma).

**Electronic supplementary material:**

The online version of this article (10.1007/s11306-019-1632-0) contains supplementary material, which is available to authorized users.

## Introduction

Androgens play a key role in embryo development and have an essential impact on the growth and development of the male reproductive system (Mooradian et al. 1987). They also exert multiple effects on different physiological processes by the activation of the androgen receptor (Matsumoto et al. 2013; Mauvais-Jarvis 2011) and subsequent transcription of targeted genes (Lee and Chang 2003). Because of the effects of the activation of the androgen receptor, synthetic anabolic androgenic steroids (AASs) are administered in the clinic (e.g. for treatment of androgen deficiency in men) (Kicman 2008). However, abuse of AASs is believed to be widespread among bodybuilders because supratherapeutic doses of synthetic AASs enhance athletic performance and physical appearance (Kanayama et al. 2010). Such high doses of AASs entail behavioral changes, cardiovascular incidents, and metabolic disorders (Grönbladh et al. 2013; Kanayama et al. 2008; Pospíšilová et al. 2012; Steensland et al. 2005; van Amsterdam et al. 2010; Zelleroth et al. 2019). Hence, the effects of supratherapeutic doses of AASs are a source of public concern.

AASs, the androgen receptor, lipid metabolism, and disease present an intertwined and complex relationship (Gårevik et al. 2011). It is known that supratherapeutic doses of AASs remodel lipoprotein metabolism in the liver by decreasing high density lipoproteins (HDL), but increasing low density lipoprotein (LDL) (Dhar et al. 2005; Niedfeldt 2018). In addition, by regulating lipid biosynthesis and energy homeostasis, the androgen receptor is involved in pathophysiological processes with dysregulated lipogenesis, such as obesity and type 2 diabetes (Mauvais-Jarvis 2011). From a mechanistic point of view, overexpression of the androgen receptor in hepatoma cells downregulates lipogenic enzymes (Kanda et al. 2017). This effect is mediated by a cross-talk between the androgen receptor and the lipogenic liver X receptors (LXRs), that inhibits the activity of the LXRs (Krycer and Brown 2011). LXRs, whose endogenous ligands are oxysterols and other cholesterol derivatives, are key regulators of de novo lipogenesis and play a key role in many diseases (e.g. type 2 diabetes, fatty liver disease, or cancer) (Jakobsson et al. 2012; Maqdasy et al. 2016; Patterson et al. 2017; Wang and Tontonoz 2018). How do LXRs regulate lipogenesis? On the one hand, LXRs increase the de novo synthesis of fatty acids via direct upregulation of fatty acid synthase (FAS) and stearoyl-CoA desaturase 1 (SCD-1) (Wang and Tontonoz 2018). On the other hand, LXRs induce, at the transcriptional level, the sterol regulatory element-binding protein 1c (SREBP-1c) and the carbohydrate-responsive element-binding protein (ChREBP) (Wang and Tontonoz 2018; Xu et al. 2013). SREBP-1c upregulates ATP-citrate lyase (ACL), acetyl-CoA synthetase (ACS), acetyl-CoA carboxylase (ACC), FAS, stearoyl-CoA desaturase 1 (SCD-1), and glycerol-3-phospate acyltransferase 1 (GPAT-1) (Xu et al. 2013). ChREBP upregulates L-type pyruvate kinase, ACC, and FAS (Xu et al. 2013). Furthermore, the androgen receptor induces de novo synthesis of fatty acids and cholesterol via SREBP-2, which upregulates HMG-CoA reductase, FAS, and LDL receptor (Horton et al. 2002). The first expected effect of the downregulation of the LXR pathway in the liver would be the decrease of the de novo synthesis fatty acids to reduce the availability of palmitic, palmitoleic, stearic, and oleic acids. In contrast with these saturated and monounsaturated fatty acids, polyunsaturated acids in the liver have their origin in the diet, either as essential fatty acids or by elongation of the essential fatty acids (Mohrhauer and Holman 1963). While saturated and monounsaturated fatty acids in the liver also originate from the diet, it seems that the main factor that rule their levels during fasting is their de novo synthesis (Balgoma et al. 2019b). Subsequently in the metabolic cascade, the downregulation of LXR in the liver would decrease the de novo synthesis of glycerolipids with palmitic, palmitoleic, stearic, and oleic acids. Finally, the downregulation of LXR in the liver would decrease the de novo synthesis of sphingolipids by the reduction of available palmitic acid. These regulations are summarized in SM-2.

Consequently, we hypothesized that the activation of the androgen receptor inhibits the activity of LXRs in the liver, which would be reflected in the profile of the plasma lipidome by the known contribution of very low density lipoproteins (VLDL) into lipoproteins (Choi and Ginsberg 2011). Briefly, we expected: (1) a decrease in plasma of glycerolipids (triacylgycerides and glycerophospholipids) with palmitic, palmitoleic, stearic, and oleic acids; and (2) a decrease in plasma of sphingolipids (SM-2). In order to test this hypothesis, we used mass spectrometry lipidomics, which has the capability of performing relative quantification and structural determination of sphingolipids and glycerolipids (Balgoma et al. 2008, 2013; Holčapek et al. 2018). While there are semiquantiative approaches to lipidomics (Tu et al. 2018), relative quantification of lipids fitted the aims of this study. For example, TG(54:5) is approximately five times more abundant than TG(50:0) in human plasma (Quehenberger et al. 2010). A priori, quantitative lipidomics would lead to neglect changes in minor species [TG(50:0) in our example]. However, in contrast to TG(54:5), minor TG(50:0) is associated with dysregulated lipid metabolism in the liver (Balgoma et al. 2019b). Hence, relative quantification fitted the purpose of this study.

In this context, the primary aim of this study was to investigate if supratherapeutic doses of AASs affected the profile of the plasma lipidome according to our hypothesis (SM-2). To the best of our knowledge, this profile has not been described before. We also aimed to profile and interpret other changes in the plasma lipidome in the context of previous research. Consequently, we treated male *Wistar* rats with supratherapeutic doses of AASs (nandrolone decanoate and testosterone undecanoate). Thereafter, we profiled the changes in the plasma lipidome by relative quantification using mass spectrometry lipidomics. The changes in the plasma lipids indicated that AASs in supratherapeutic doses suppressed LXR-mediated de novo lipogenesis in the liver, which agrees with our hypothesis (SM-2).

## Materials and methods

### Animal treatment

All animal experiments were performed in accordance with the guidelines of the Swedish Legislation on Animals Experimentation (Animal Welfare Act SFS 1998:56) and the European Union Directive on the Protection of Animals Used for Scientific Purposes (2010/63/EU). The procedures included in the study were approved by the local Uppsala animal ethics committee (5.8.18-02249/2017). Thirty-six male *Wistar* rats (Envigo, Netherlands), seven weeks old at arrival, were used in the study. The animals were housed in groups of three in standard cages type IV (59 × 38 × 20 cm, with elevated lids), under standardized housing conditions (i.e. 20–24 °C and a humidity of 45–65%), and on a reversed 12 h dark/light cycle (lights on at 6 pm). To adjust to the new environment the animals were allowed 14 days of acclimatization before the start of the experiments. Food (standard pellet type R36, Lantmännen, Kimstad, Sweden) and water were provided ad libitum. The animals were monitored daily, and weighed regularly throughout the study period. Nandrolone decanoate (Deca-durabol®) was manufactured by Organon (Netherlands), testosterone undecanoate (Nebido®) was produced by Bayer AG (Germany), and the peanut oil was obtained from Apl (Sweden). The animals were randomized into three treatment groups (12 animals per group), and were administered either 15 mg/kg of nandrolone decanoate (50 mg/mL in peanut oil), 15 mg/kg testosterone undecanoate (50 mg/mL in peanut oil and castor oil, 80:20 v/v), or vehicle (peanut oil). All animals received subcutaneous injections on the upper back in volumes of 100 µL every third day throughout the study (days 1–18, six injections per animal in total). On day 18, the animals were euthanized by decapitation and trunk blood was collected in lithium-heparin treated collection tubes (Sarstedt, Sweden). Thereafter, the blood was centrifuged for 10 min at 1500×*g* in 4 °C, and subsequently plasma fractions were collected and stored at − 80 °C for further analysis.

### Lipid profiling

The amount of sample extracted was optimized for the relative quantification of major species in plasma. The lipid content of plasma was isolated by single phase precipitation of protein (modified from Satomi et al. (2017)). Briefly, 200 µL of acetonitrile/isopropanol (1:1, v/v) were added to 20 µL of plasma in a microtube. Subsequently, the samples were vortexed for 15 s and incubated under agitation at room temperature for 1 h. After centrifugation at 10,000 rpm for 5 min, the supernatant was isolated for injection. A quality control sample was made as a pool of aliquots of every extract.

Lipids in the extract were separated on an Acquity-UPLC (Waters) with a BEH C18 column (1.7 µm, 2.1 × 150 mm) at 55 °C and a gradient of solvents A water/acetonitrile/isopropanol 40:30:30 (v/v/v) with 5 mM of ammonium formate, and B acetonitrile/isopropanol 40:60 (v/v) with 5 mM of ammonium formate. The gradient (flow 0.275 ml min^−1^) changed linearly from 95% of A at min 0, to 77% at min 3.25, to 45% at min 3.5, to 43% at min 6, to 32% at min 6.25, to 29% at min 9.5, to 9% at min 9.75, and to 1% at min 13, which was kept until min 16. The eluent was ionized by electrospray on a Synapt G2S Q-ToF (Waters) in positive and negative mode scanning between *m/z* 100 and 1500. Both extraction and injection of samples were randomized to avoid analytical biases. The quality control sample was injected every seven injections of samples. The composition of fatty acids (number of carbons and unsaturations) of glycerolipids was characterized by fragmentation experiments (MS^2^, SM-1) (McAnoy et al. 2005; Pulfer and Murphy 2003). When more than one combination of fatty acids was possible, a peak was assigned to the combination of fatty acids with the highest signal (“Main species” column in SM-1).

### Data pre-treatment

Water’s Raw mass spectrometry files were transformed with Data Bridge into CDF files and processed with XCMS. To identify the lipids, a database of lipids from Lipid Maps was used (Fahy et al. 2009). Subsequently, the m/z values of the parent ions and the fragments were compared with an in-house database of lipids built from the molecular formulae of the adducts by package Rdisop in R. The lipid signal was quantified as the area under the chromatographic curve of the selected peaks. Subsequently, relative quantification (normalized signal) was performed by measuring the ratios between the signals obtained from the study samples and the signals of the quality control sample (pool of samples).

### Data analysis

While many lipidomics studies focus on the description of “statistically significant” changes, we focused this study on the biological interpretation of the changes of lipids in the context of previous research. This strategy approaches the problems of dichotomous cutoffs for p-values (Betensky 2019), which have recently been highlighted in an editorial by Wasserstein et al. (2019) in The American Statistician following the statement of the American Statistical Association on p-values (Wasserstein and Lazar 2016). Confidence intervals and fold changes were used instead of p-values, as recommended by Gardner and Altman (1986).

Univariate analysis of the signals of the lipids was performed by the analysis of the confidence intervals of the fold changes to the control group. The fold change of a lipid was quantified as the natural logarithm of the ratio between the average of the normalized signal from a sample from a treated individual and the average of the signal in the control group, i.e. log(Lipid_treatment_/Lipid_control_) (0 means no change). The fold change of the weight for a specific animal was quantified as the natural logarithm of the ratio between the weight at the ending of the assay (day 18) and the weight before the treatment (day 0), i.e. log(W_animal,day 0_/W _animal,day 18_) (0 means no change). In all cases, the intervals of confidence of the fold changes of the averages were calculated by resampling 10,000 times by bootstrap method and selecting the percentiles at 2.5% and 97.5% as lower and upper limits (James et al. 2013).

Multivariate analysis of the signals of the lipids was performed by principal component analysis (James et al. 2013) after transforming by the natural logarithm, centering by the average, and scaling by the standard deviation. The relationship between the treatment with AASs and the principal components was studied by the scores. The correlation and clustering among lipids was studied by the loadings.

LION/Web was used to study the lipid ontology of clusters of variables in the first two components (SM-3) (Molenaar et al. 2019).

### Software

Data pre-treatment and analysis were performed on R 3.4.3 (*Kite-Eating Tree*) run under RStudio 1.1.383. R packages XCMS 3.4.4 and Rdisop 1.42.1 were used. Figures were created with R 3.4.3, GIMP 2.10.8, and Inkscape 0.92.

## Results

### Effect of AASs on the fold changes of individual lipids: univariate analysis

**Fig. 1:**
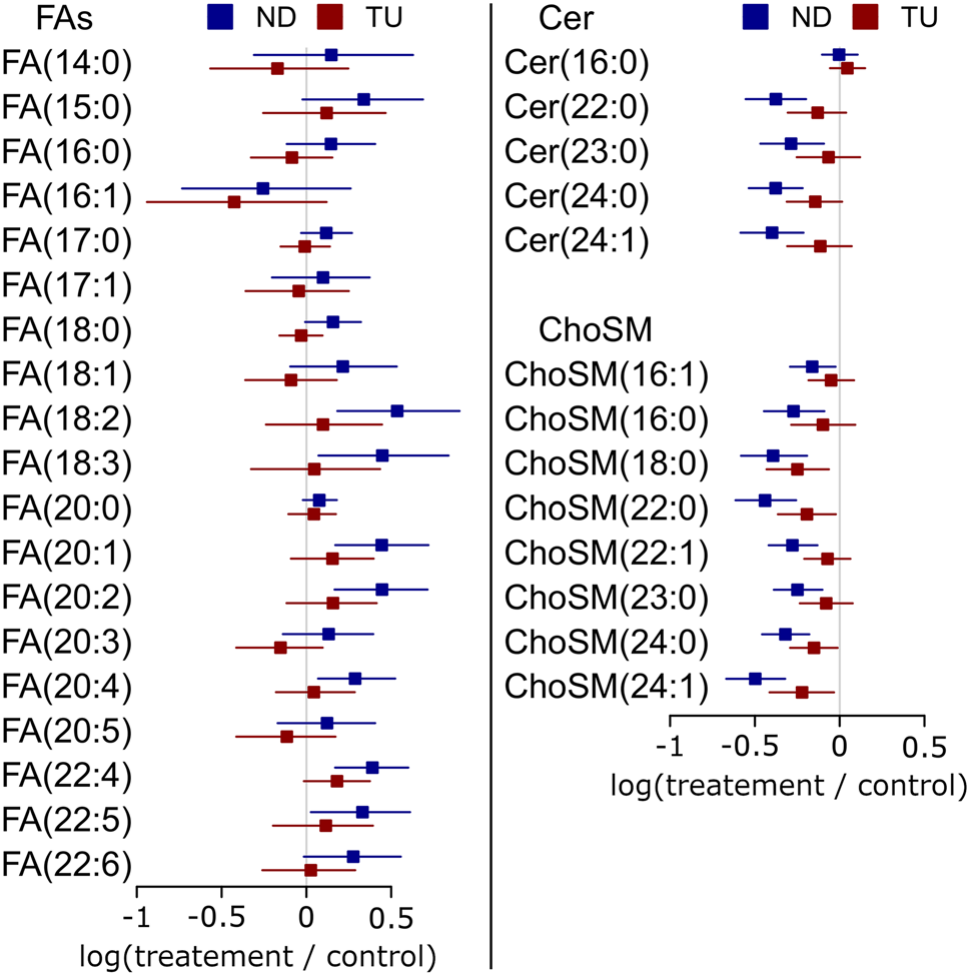
Fold changes of the free fatty acids (FAs), ceramides (Cer), and sphingomyelins (ChoSM) in plasma. The average of the fold changes of the treatments is represented by squares: blue squares for nandrolone decanoate (ND) and red squares for testosterone undecanoate (TU). The confidence intervals by bootstrap resampling are represented by the horizontal bars

Nandrolone decanoate and testosterone undecanoate exerted different effects on free fatty acids in plasma (FAs in Fig. 1). Except for FA(16:1), nandrolone decanoate exhibited a trend to increase the concentration free fatty acids in plasma, as the averages of the fold changes were positive (Fig. 1). This indicates that the increase provoked by nandrolone decanoate on plasma free fatty acids is general and not specific for any lipid. On the contrary, testosterone undecanoate did not exert a clear effect on free fatty acids. For example, linoleic acid [FA(18:2)] showed a tendency to increase with testosterone undecanoate, but oleic acid [FA(18:1)] showed a tendency to decrease (Fig. 1).

Regarding sphingolipids, both ceramides (Cer) and sphingomyelins (ChoSM) presented a common trend to decrease with both nandrolone decanoate and testosterone undecanoate (Fig. 1).

**Fig. 2:**
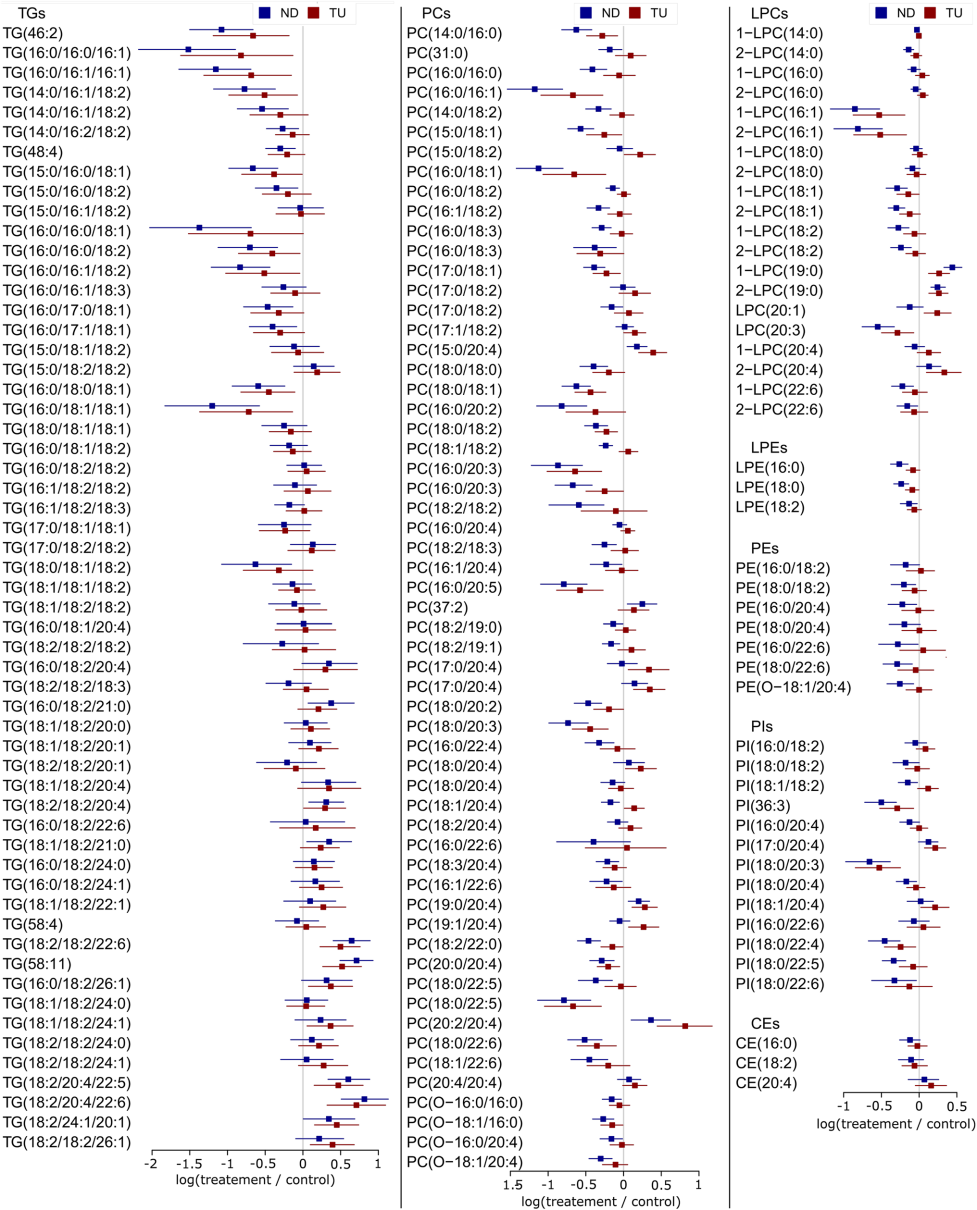
Fold changes of the glycerolipids and cholesteryl esters in plasma. The average of the fold changes of the treatments is represented by squares: blue squares for nandrolone decanoate (ND) and red squares for testosterone undecanoate (TU). The confidence intervals by bootstrap resampling are represented by the horizontal bars

The different species of triacylglycerides (TGs in Fig. 2) displayed a high variability in their behavior. The species with low number of carbons and unsaturations decreased with both nandrolone decanoate and testosterone undecanoate. Specifically, the TGs with palmitic, palmitoleic, and oleic acids showed the strongest downregulation: TG(16:0/16:0/16:1), TG(16:0/16:1/16:1), TG(16:0/16:0/18:1), and TG(16:0/18:1/18:1). However the species with high number of carbons and unsaturations increased with both treatments (e.g. TG(18:2/18:2/22:6) and TG(18:2/20:4/22:6)). These triacylglycerides contained highly unsaturated fatty acids, such as arachidonic acid (acyl chain 20:4) and docosahexaenoic acid (acyl chain 22:6).

Regarding lysoglycerophospholipids of choline (LPCs, Fig. 2), the lipid species with palmitoleic acid (1-LPC(16:1), and 2-LPC(16:1) in Fig. 2) displayed the strongest decreases with both treatments. The glycerophospholipids of choline (PCs) exhibited two opposite patterns of regulation. Several species with palmitic, palmitoleic, stearic, or oleic acids (Fig. 2) showed a strong downregulation with both treatments: PC(16:0/16:1); PC(18:0/18:1). This behavior agrees with the trend of triacylglycerides with saturated and monounsaturated fatty acids (Fig. 2). In contrast, several species increased with both treatments (Fig. 2): PC(17:1/18:2), PC(15:0/20:4), PC(37:2), PC(17:0/20:4), PC(18:0/20:4), PC(19:0/20:4), PC(20:2/20:4), and PC(20:4/20:4). The fatty acid composition of this group of lipids indicates the upregulation of odd-numbered chain fatty acids (e.g. margaric acid, acyl chain 17:0) and arachidonic acid (acyl chain 20:4).

The glycerophospholipids of ethanolamine (PEs in Fig. 2) contained a combination of a saturated fatty acid and a polyunsaturated fatty acid (e.g. PE(16:0/22:6)). In contrast with the glycerophospholipids of choline (PCs), the different glycerophospholipids of ethanolamine presented a trend to decrease with nandrolone decanoate, but no clear trend with testosterone undecanoate. The lysoglycerophospholipids of ethanolamine (LPEs in Fig. 2) behaved similarly.

The glycerophospholipids of inositol (PIs in Fig. 2) contained a saturated or monounsaturated fatty acid and a polyunsaturated fatty acid. Among them, PI(18:0/20:3) showed the deepest decrease. However, PI(17:0/20:4) and PI(18:1/20:4) showed a tendency to increase. This behavior agrees with the upregulation of odd-numbered chain fatty acids and arachidonic acid in glycerophospholipids of choline (Fig. 2).

### Effect of AASs on the plasma lipidome: multivariate analysis

**Fig. 3:**
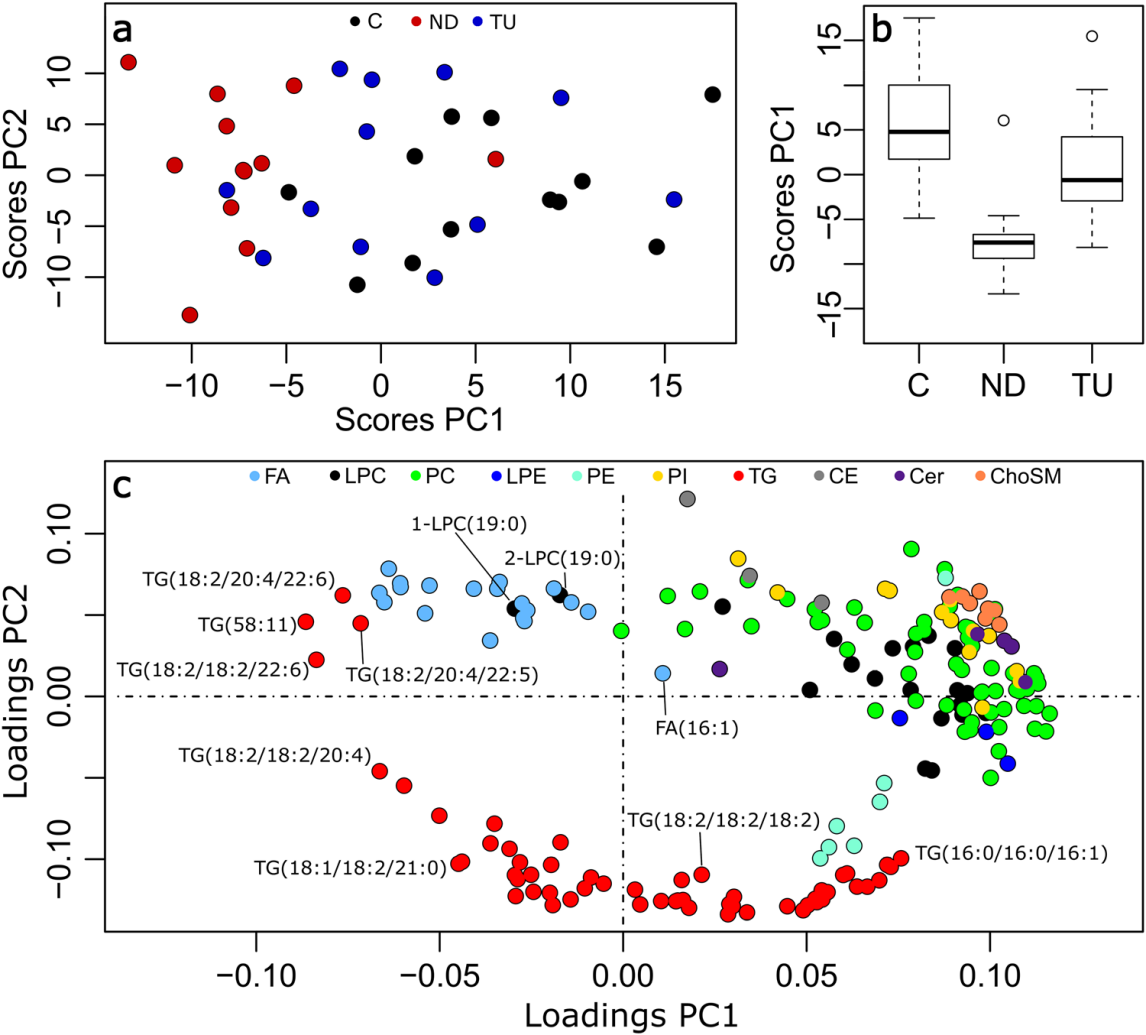
Principal component analysis of the lipidome. **a** Scatter plot of the scores of the first two principal components (PC) by treatment: control (C, black), nandrolone decanoate (ND, red), testosterone undecanoate (TU, blue). **b** Boxplots of the scores of the first principal component by the treatment. **c** Loading plot of the first two principal components by family of lipids

The first two principal components described the major changes in the lipidome in relation to the treatment with nandrolone decanoate and testosterone undecanoate (explaining, respectively, 33 and 24% of the variance, Fig. 3a). The nandrolone decanoate group showed good separation from the control group by the scores of the first principal component (Fig. 3b). However, the testosterone undecanoate group showed minor separation.

The comparison of the loadings of the first two principal components indicated that most lipids correlated with the first principal component (i.e. on the right of the scatterplot of Fig. 3c).

Triacylglycerides and free fatty acids differed from this general pattern. Triacylglycerides spread along the first principal component and correlated negatively with the second principal component (Fig. 3c). Four triacylglycerides clustered in the fourth quadrant of Fig. 3c (i.e. negative first principal component and positive second principal component): TG(18:2/18:2/22:6), TG(58:11), TG(18:2/20:4/22:5), and TG(18:2/20:4/22:6). This statistical behavior suggests specific regulation of the triacylglycerides with highly unsaturated fatty acids.

Free fatty acids (FAs) also clustered in the fourth quadrant of Fig. 3c, which indicates that free fatty acids do not follow the general pattern of decrease of the concentration lipids in plasma with the treatment with AASs. As detected in univariate analysis (Fig. 1), FA(16:1) did not cluster with the rest of the free fatty acids.

Among lysoglycerophospholipids of choline, 1-LPC(19:0) and 2-LPC(19:0) did not cluster with the rest of the species. This behavior suggests specific regulation of odd-numbered chain fatty acids and is in accordance with the univariate analysis.

As a final observation, the loadings of the sphingolipids behaved remarkably. While other lipid families (e.g. triacylglycerides), spread along a wide range of loadings, ceramides and sphingomyelins clustered together in a narrow range of loadings (Cer in purple and ChoSM in orange in Fig. 3c). This fact suggests a common point of regulation that acts as a bottleneck in the regulation of sphingolipids. This common point of regulation would induce a high correlation in the levels of sphingolipid in the liver and plasma, which is reflected as a cluster in the loadings of the principal components.

## Discussion

**Fig. 4:**
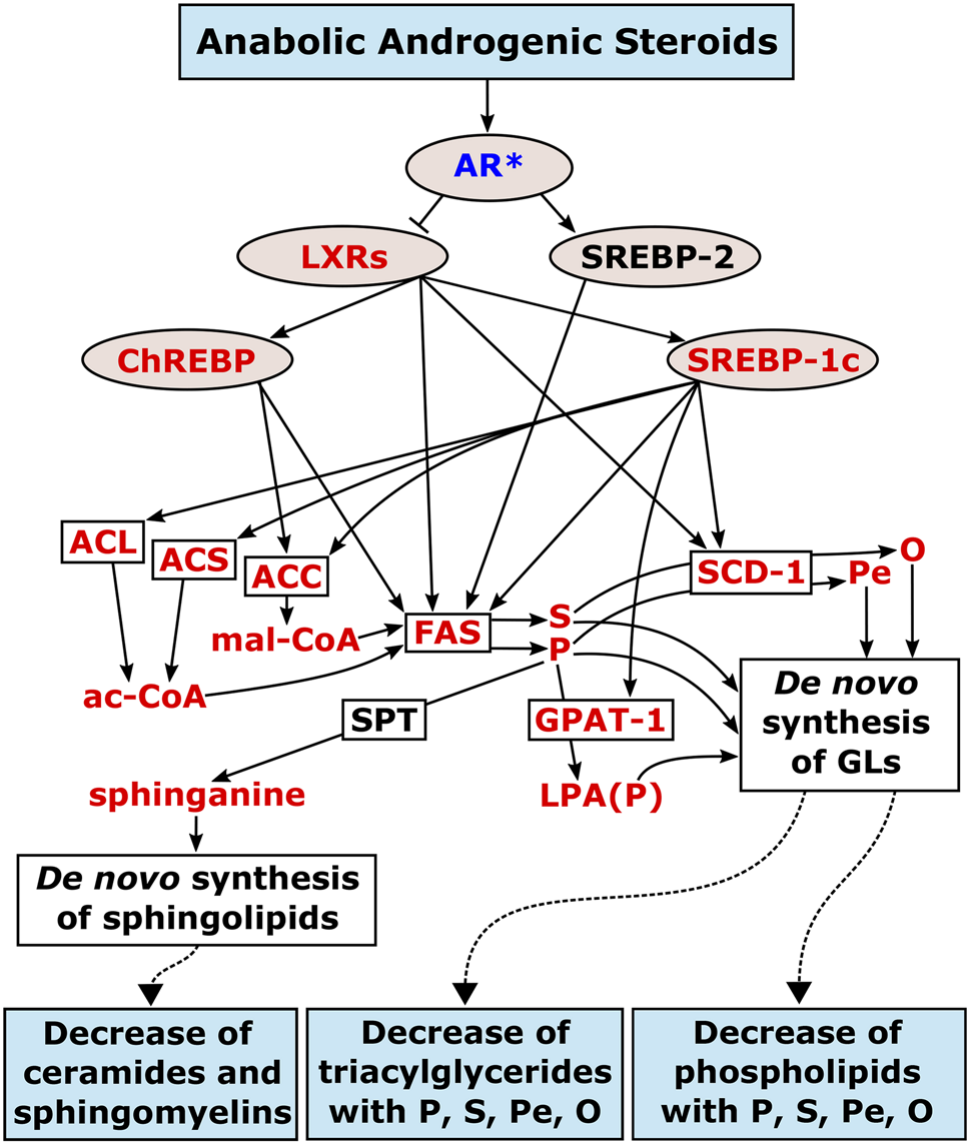
Proposed relationship between AASs, de novo lipogenesis (fatty acids, glycerolipids, and sphingolipids), and changes in the plasma lipidome in the light of prior knowledge. Blue boxes represent the treatment and observations in the plasma lipidome. The elements in between represent the regulatory points and enzyme activity that relate the treatment and the observed changes in the plasma lipidome. Light brown ovals represent regulatory elements. White boxes represent lipogenic enzymes or pathways. The metabolites are represented without contour. Red color indicates downregulation, blue color upregulation. Abbreviations: AR, androgen receptor; ac-CoA, acetyl coenzyme A; mal-CoA, malonyl coenzyme A; S, stearic acid; P, palmitic acid; O, oleic acid; Pe, palmitoleic acid; LPA(P), 1-palmitoyl-3-phosphoglycerol; GLs, glycerolipids

Here we describe for the first time how AASs affect the profile of the plasma lipidome. Considering the primary goal of this study, the changes of glycerolipids and sphingolipids matched the hypothesis described in SM-2. Consequently, these results suggest that AASs decrease de novo lipogenesis by the downregulation of the activity of the LXR pathway via activation of the androgen receptor (Fig. 4). In addition, a general decrease of free fatty acids but an increase glycerolipids with odd-numbered chain fatty acids and/or polyunsaturated fatty acids was observed. These changes, might partially explain the role of AASs and androgens in cardiovascular and metabolic diseases.

### Glycerolipids and sphingolipids in plasma mirror the downregulation of the LXR-mediated lipogenesis in the liver

The triacylglycerides with palmitic, stearic, palmitoleic, and oleic acids showed a more pronounced decrease than the other triacylglycerides (Fig. 2). This profile matched the expected changes by the hypothesized downregulation of LXRs and subsequent downregulation of ACL, ACS, ACC, FAS, SCD-1, and GPAT-1 (Fig. 4). Despite the hypothetical nature of this relationship, it follows the principle of parsimony in the context of previous research.

Regarding glycerophospholipids in plasma, it should be considered that their regulation is more complex than that of triacylglycerides. In the liver, in addition to the de novo synthesis, phosphatidylethanolamine *N*-methyltransferase (PEMT) converts glycerophospholipids of ethanolamine into glycerophospholipids of choline (Vance 2014). This pathway accounts for 30% of the glycerophospholipids of choline in the liver. Nowadays, it is considered that PEMT is not selective for the fatty acids in the glycerophospholipids of ethanolamine (Vance 2014). In addition, lecithin-cholesterol acyltransferase (LCAT) transfers HDL fatty acids from the sn-2 position of glycerophospholipids of choline to cholesterol in plasma. This activity yields most of the cholesteryl esters in plasma (Lima 2004; Ossoli et al. 2016). Consequently, the activity of PEMT and LCAT makes the interpretation of their regulation more complex than in the case of triacylglycerides. Nevertheless, the species with combinations of palmitic, stearic, palmitoleic, and oleic acids displayed the strongest decreases in glycerophospholipids of choline (Fig. 2). This profile also matched the expected changes by the downregulation of LXRs and subsequent downregulation of ACL, ACS, ACC, FAS, and SCD-1 in the de novo synthesis of fatty acids, and GPAT-1 in the de novo synthesis of glycerophospholipids (Fig. 4). Different studies have investigated the de novo synthesis of glycerophospholipids by means of isotope-labeled headgroups (Balgoma et al. 2010a, b; Brandsma et al. 2017; DeLong et al. 1999; Ecker and Liebisch 2014; Postle et al. 2004). Specifically, previous studies in hepatocytes have shown that the species with combinations of palmitic, palmitoleic, stearic, and oleic acids are the main products of the hepatic de novo synthesis of glycerophospholipids of choline (DeLong et al. 1999). Despite putative confounding factors, it seems plausible in this context to address the strongest decreases of glycerophospholipids in plasma (Fig. 2) to the decrease of their de novo synthesis in the liver.

Ceramides and sphingomyelins presented a homogeneous decrease and correlated decrease in both univariate and multivariate analysis (Figs. 1 and 3c). This behavior suggests a common point of regulation in the first steps of the de novo synthesis of sphingolipids in the liver. In the light of previous knowledge (Cowart and Hannun 2007), the availability of FAS-synthesized palmitic acid in the liver would act as a bottleneck in the synthesis of sphinganine, the building block of sphingolipids. Again, the behavior of sphingolipids matched the expected changes by the downregulation of sphinganine (Fig. 4) (Cowart and Hannun 2007).

Finally, we would like to highlight that LXR-regulated GPAT-1 transfers palmitic acid in a selective way to the sn-1 position of glycerol-3-phosphate (Table S.1). Other GPATs are not selective nor regulated. Consequently, GPAT-1 controls not only the content of palmitic acid, but also the position of palmitic acid in glycerolipids. Thus, we suggest that the regioisomeric and/or enantiomeric analysis of the glycerolipids in plasma is important to understand the contribution of GPAT-1 to de novo synthesis of glycerolipids in future clinical studies (Balgoma et al. 2019a, b).

### Upregulation of glycerolipids with highly unsaturated and odd-numbered chain fatty acids: potential effect of the aromatization of AASs

AASs increased different glycerolipids with highly unsaturated fatty acids (e.g. TG(18:2/18:2/22:5), TG(18:2/20:4/22:6), TG(18:2/20:4/22:5), LPC(20:4), PC(20:2/20:4), and PC(20.4/20:4) in Fig. 2). Several previous studies have related the glycerolipids with highly unsaturated fatty acids to estrogens. For example, PC(16:0/20:5), and TG(62:12) are upregulated in the blood plasma from women in comparison with men (Maekawa et al. 2017). Highly unsaturated triacylglycerides and PC(40:8) (putatively PC(20:4/20:4) (Balgoma et al. 2010a, b)) are upregulated in a model of estrogen-related endometriosis (Dutta et al. 2016). Tamoxifen (an inhibitor of estrogen effect) decreases triacylglycerides with highly unsaturated fatty acids in the liver and in plasma (Saito et al. 2017). Furthermore, it is known that estrogen receptor 2 upregulates the synthesis of arachidonic acid (Ariazi et al. 2011). This effect of estrogens is mediated by fatty acids desaturases (FADSs) (Ariazi et al. 2011; Saito et al. 2017). In this context, it seems plausible that the supratherapeutic doses of nandrolone decanoate and testosterone undecanoate in our study activate the estrogen receptor after aromatization (Simpson et al. 2002). Subsequently, the activation of the estrogen receptors would upregulate FADSs and increase the availability of highly unsaturated fatty acids for the synthesis of glycerolipids. These fatty acids would be incorporated into the different glycerolipids by different acyltransferases (Tijburg et al. 1989).

Different glycerophospholipids with odd-numbered chain fatty acids also increased [e.g. PC(15:0/20:4), PI(17:0/20:4)], which suggests the upregulation of these fatty acids. We had not hypothesized this change, but several previous studies have related odd-numbered chain fatty acids to estrogens. For example, PC(17:0/20:4) is higher in the plasma from women than in the plasma from men (Maekawa et al. 2017). Consistently, tamoxifen also decreases the glycerophospholipids with odd-numbered chain fatty acids (Saito et al. 2017). While traditionally associated with food intake, odd-numbered chain fatty acids are also synthesized by the elongation of propionate from the catabolism of amino acids (Crown et al. 2015; Pfeuffer and Jaudszus 2016). Similarly to highly unsaturated fatty acids, in the context of previous research, it seems plausible that supratherapeutic doses AASs activate the estrogen receptor after aromatization, which would lead hypothetically to the upregulation of propionate, the precursor of odd-numbered chain fatty acids.

Against these putative increases of polyunsaturated fatty acids and odd-numbered chain fatty acids, it could be argued that some glycerophospholipids with these fatty acids present minor relative changes [e.g. PC(18:0/20:4), PC(17:0/18:1), PE(18:0/20:4), and PI(16:0/22:6)]. In addition, some of these species even present a decrease [e.g. PC(15:0/18:1), PC(18:0/22:6), and PI(18:0/22:4)]. These species have (1) one palmitic, stearic or oleic acid, and (2) one polyunsaturated or odd-numbered chain fatty acid. As discussed in Sect. 4.1, palmitic, stearic and oleic acids would be strongly downregulated in the liver. Under the assumption of the upregulation of polyunsaturated fatty acids and odd-numbered chain fatty acids, the availability of the building fatty acids of these “mixed” species follow opposite directions: one fatty acids is less available, but the other fatty acid is more available. We interpret the change of these “mixed” species as a balance between these two contradictory availabilities. This behavior would lead to minor changes (or even decreases) of these “mixed” species of glycerophospholipids. Consequently, the contradiction between the behavior of these “mixed” species of glycerophospholipids and the proposed upregulation of polyunsaturated and odd-numbered chain fatty acids is only apparent.

In conclusion, the profile of changes of the plasma lipidome (Fig. 2) after treatment with AASs is compatible with the upregulation of polyunsaturated and odd-numbered chain fatty acids. Previous research suggests—in an indirect way—that these upregulations are related to the levels of estrogens. In the context of previous research, it seems plausible that the increase of these species are related with the aromatization of AASs. Further metabolic research is warranted to unravel the relationship among androgen aromatization, estrogens, and both polyunsaturated and odd-numbered chain fatty acids.

### Analysis of the lipid ontology by LION/web: upregulation of free fatty acids and lipolysis in the adipose tissue

To complement the previous discussion, we used a recently published tool to enrich the dataset in lipid ontology (Molenaar et al. 2019). The ontology of the clusters of lipid variables agreed with our previous discussion (SM-3).

Ontological palmitic (C16:0, LION:0002882), palmitoleic (C16:1, LION:0002900), and oleic (C18:1, LION:0002922) acids clustered (SM-3). The visual inspection of the heatmap shows decreased levels for the AAS-treated groups. This behavior agrees with Fig. 2 and the discussion in Sect. 4.1.

In addition, ontological arachidonic (C20:4, LION:0002929) and docosahexaenoic (C22:6, LION:0002937) acids also clustered together with other long chain polyunsaturated fatty acids (SM-3). The visual inspection of the heatmap suggests an increase in the group treated with testosterone undecanoate, which agrees with the discussion of Fig. 2 in Sect. 4.2. These clusters reinforce our suggestion of an upregulation of fatty acids desaturases [despite the apparent contradiction with the decrease of species such as PC(18:0/22:6)].

Ontological nonadecanoic acid (C19:0, LION:0022241) showed a specific cluster. Visual inspection of the heatmap suggests upregulation of this odd-numbered chain fatty acid in the groups treated with AASs, which agrees with the discussion in Sect. 4.2.

Finally, free fatty acids (fatty acids [FA], LION:0000093) clustered separately from the rest of the lipids, which was also suggested in the principal component analysis (Fig. 3). Visual inspection of the heatmap suggests the upregulation of free fatty acids in the plasma of the group treated with nandrolone decanoate. This observation agrees with the behavior in Fig. 1. This fact is in apparent contradiction with the downregulation of palmitic, palmitoleic, stearic, and oleic fatty acids discussed in Sect. 4.1. Nevertheless, free fatty acids in plasma do not originate from the liver but reflect the level of lipolysis in adipose tissue (Capurso 1991; Hellmuth et al. 2013). From a data analysis point of view, this different origin explains why free fatty acids do not cluster with the rest of the lipids in multivariate analysis (Fig. 3c, SM-3).

Consequently, we address the increase of fatty acids to an increase of lipolysis in the adipose tissue by nandrolone decanoate. This higher lipolysis suggested by the increase of free fatty acids would be associated with the lack of weight gain in the rats treated with nandrolone decanoate (Grönbladh et al. 2013; Hallberg et al. 2000, 2005). Previous research suggests that the increase of lipolysis in adipose tissue by AASs could be addressed by two factors (or a combination of them): (1) a direct metabolic effect of androgens on lipolysis (Mauvais-Jarvis 2011), (2) a decrease of food intake (Lindblom et al. 2003). Furthermore, the decrease of food intake would also downregulate SREBP-1c in the liver by a decrease of insulin levels. Consequently, the effect of AASs on diet and indirectly on insulin levels might also explain the regulation of the plasma lipids discussed in Sect. 4.1 (Maqdasy et al. 2016; Wang and Tontonoz 2018; Xu et al. 2013).

### Relationship between the changes in the plasma lipidome and disease

In relation to cardiovascular risk, some of the observed changes in the plasma lipidome seem to be protective. For example, the sphingolipids and triacylglycerides with low number of carbons and unsaturations in plasma decreased after administration of supratherapeutic doses of AASs. These lipids are positively correlated with cardiovascular diseases, which suggests a better profile (Havel 2010; Jiang et al. 2000; Stegemann et al. 2014). In addition, we observed an increase in odd-numbered chain fatty acids in plasma phospholipids, which is associated with lower risk of coronary heart disease (Khaw et al. 2012). However, the increase of arachidonic acid in glycerophospholipids can be associated with higher cardiovascular disease risk through the production of eicosanoids (Funk 2001). Regarding previous research about these lipid mediators and AASs, testosterone propionate increases plasma thromboxane A_2_, but not the vasodilator prostacyclin in animal models (Lundström et al. 2011; Weyrich et al. 1992). This imbalance would increase the risk of cardiovascular disease. Consequently, the imprint of AASs in supratherapeutic doses on the plasma lipidome presents a Janus role in relation to cardiovascular risk.

In relation to metabolic diseases with upregulated lipogenesis, the changes of the plasma lipidome that we present suggest an important role of sexual hormones. While the secondary effects of supratherapeutic doses of AASs are not acceptable in patient treatment, AASs induced a decrease of triacylglycerides with palmitic, palmitoleic, stearic, and oleic acids. This group of triacylglycerides is associated with dysregulated liver metabolism in type 2 diabetes, fatty liver disease, and hepatocellular carcinoma (Balgoma et al. 2019a, b). It is known that these diseases present upregulation of LXRs (Wang and Tontonoz 2018). Consequently, in a broad perspective, the profile of lipids that we present will help to understand the influence of androgens in diseases with dysregulated lipogenesis and overexpression of LXRs in future studies. For example, it is known that the activation of the androgen receptor decreases lipogenesis in type 2 diabetes, which is associated with lower insulin resistance (Mauvais-Jarvis 2011). Therefore, the profile of plasma lipids that we present helps to understand the interplay among androgens, lipid metabolism, and insulin resistance in type 2 diabetes.

## Conclusions

To the best of our knowledge, our work reveals for the first time the fingerprint of AASs on individual lipid species in the plasma lipidome. In this profile, AASs induce a decrease of glycerolipids with palmitic, palmitoleic, stearic, and oleic acids together with a general decrease of sphingolipids. In the light of previous research, this profile suggests the downregulation of LXR-mediated de novo synthesis of glycerolipids and sphingolipids in the liver by the activation of the androgen receptor. These results highlight the capacity of lipidomics in endocrinology studies to characterize the activity of the LXR pathway, which is present in the central nervous system, the liver, reproductive system, and hormone-related cancer (Maqdasy et al. 2016; Shackleford et al. 2017; Wang and Tontonoz 2018). In conclusion, lipidomics profiling emerges as a key discipline in the study of the interaction among sex hormones, lifestyle, and LXRs in de novo lipogenesis in health and disease.

## Supplementary Material

SM-1 contains the LC–MS characterization of lipids, the fold changes of the identified lipids, and the loadings of the lipids in principal component analysis. SM-2 contains the description of the previous research that relates the AASs, the de novo lipogenesis in the liver, and the profile of lipids in blood plasma. SM-3 contains the analysis by LION/Web lipid ontology tool.

## Electronic supplementary material

Below is the link to the electronic supplementary material.

Supplementary file1 (XLSX 79 kb)

Supplementary file2 (DOCX 200 kb)

Supplementary file3 (SVG 1010 kb)

## Data Availability

The raw lipidomics data in this paper are available via https://github.com/dbalgoma study identifier AAS_plasma-lipidomics.
