# Supplementary material for: Anabolic androgenic steroids exert a selective remodeling of the plasma lipidome that mirrors the decrease of the de novo lipogenesis in the liver
Source: Metabolomics. 2020 Jan 10;16(1):12. doi: 10.1007/s11306-019-1632-0 (PMC6954146; doi:10.1007/s11306-019-1632-0)
Supplement: Supplementary file 2 — Supplementary file2 (DOCX 200 kb) [file 11306_2019_1632_MOESM2_ESM.docx]

**Anabolic androgenic steroids exert a selective remodeling of the plasma lipidome that mirrors the decrease of the *de novo* lipogenesis in the liver**

**Supplementary Material 2: Previous research on anabolic androgenic steroids, lipogenesis in the liver, and the lipid profile in blood plasma**

**Authors**

David Balgoma^a,*^, Sofia Zelleroth^b^, Alfhild Grönbladh^b^, Mathias Hallberg^b^, Curt Pettersson^a^, Mikael Hedeland^a^

**Affiliation**

^a^ Analytical Pharmaceutical Chemistry, Department of Medicinal Chemistry, Uppsala University, Sweden.

^b^ The Beijer Laboratory, Biological Research on Drug Dependence, Department of Pharmaceutical Biosciences, Uppsala University, Sweden

As described in the introduction, the liver expresses the androgen receptor and LXRs (Mauvais-Jarvis 2011; Wang and Tontonoz 2018). It has also been reported that the androgen receptor inhibits the activity of LXRs (Krycer and Brown 2011). Consequently, we hypothesized that the AAS-induced downregulation of lipogenic enzymes in the liver would be reflected in plasma lipidome by the contribution to plasma lipoproteins of VLDL (Choi and Ginsberg 2011). This profile has not been described before. Three synthetic pathways are involved in LXR-mediated lipogenesis: 1) the *de novo* synthesis of fatty acids, 2) the *de novo* synthesis of glycerolipids, and 3) the *de novo* synthesis of sphingolipids. Due to the enzyme selectivity in these pathways (Table S.1), a specific profile of changes was expected, which is described in the following paragraphs and in Figure S.1.

Regarding the *de novo* synthesis of fatty acids (Figure S.1), the downregulation of ACL and ACS decreases the availability of acetyl-CoA (Mathews and Mathews-Van Holde-Appling-Anthony-Cahill 2013). Furthermore, acetyl-CoA is the substrate of ACC, which yields malonyl-CoA (Mathews and Mathews-Van Holde-Appling-Anthony-Cahill 2013). Both acetyl-CoA and malonyl-CoA are the substrates of FAS. FAS yields palmitic acid as main product, but it also yields stearic acid as minor product (Smith et al. 2003). In addition, SCD-1 synthesizes palmitoleic and oleic acids from, respectively, palmitic and stearic acids (Mauvoisin and Mounier 2011). Consequently, it is expected that AASs would decrease the availability of palmitic, palmitoleic, stearic, and oleic acids in the liver.

Regarding the *de novo* synthesis of glycerolipids (Figure S.1), GPATs acylate the *sn*-1 position of glycerol-3-phosphate as a first step (Tijburg et al. 1989). Among GPATs, GPAT-1 –which is regulated by the LXR pathway (Yu et al. 2018)– shows preference for palmitic acid (Monroy et al. 1972). By this effect and the downregulation of the *de novo* synthesis of fatty acids (see previous paragraph, Figure S.1), it is expected a decrease of triacylglycerides and glycerophospholipids with palmitic, stearic, palmitoleic, and oleic acids.

Regarding the *de novo* synthesis of sphingolipids (Figure S.1), serine C-palmitoyltransferase (SPT) uses palmitic acid as substrate to yield sphinganine as a first step (Harayama and Riezman 2018). Considering that FAS-synthesized palmitic acid limits the synthesis of the sphingoid base in yeast (Cowart and Hannun 2007), it is expected that the downregulation of FAS would entail a decrease of sphingolipids.

**Table S1. Enzyme selectivity of the lipogenic enzymes in the LXR pathway: ATP-citrate lyase (ACL), acetyl-CoA synthetase (ACS), acetyl-CoA carboxylase (ACC), fatty acid synthase** (**FAS), stearoyl-CoA desaturase 1 (SCD-1), and glycerol-3-phospate acyltransferase 1 (GPAT-1).**

| **Enzyme** | **Selectivity** | **Lipids affected** |
| --- | --- | --- |
| ACL | It yields acetyl-CoA | Free fatty acids, glycerolipids, sphingolipids |
| ACS | It yields acetyl-CoA | Free fatty acids, glycerolipids, sphingolipids |
| ACC | It yields malonyl-CoA | Free fatty acids, glycerolipids, sphingolipids |
| FAS | It yields palmitic acid as major product from acetyl-CoA and malonyl-CoA. It also yields stearic acid as minor product | Free fatty acids, glycerolipids, sphingolipids |
| SCD-1 | It yields palmitoleic and oleic acids from palmitic and stearic acids, the products of FAS | Free fatty acids, glycerolipids |
| GPAT-1 | It transfers saturated fatty acids (such as palmitic and stearic acids) to the sn-1 position of glycerol-3-phosphate | Glycerolipids |

In summary, after treating *Wistar* rats with AASs we expected in plasma: 1) a decrease of triacylgycerides and glycerophospholipids with palmitic, palmitoleic, stearic, and oleic acids; and 2) a decrease of sphingolipids.


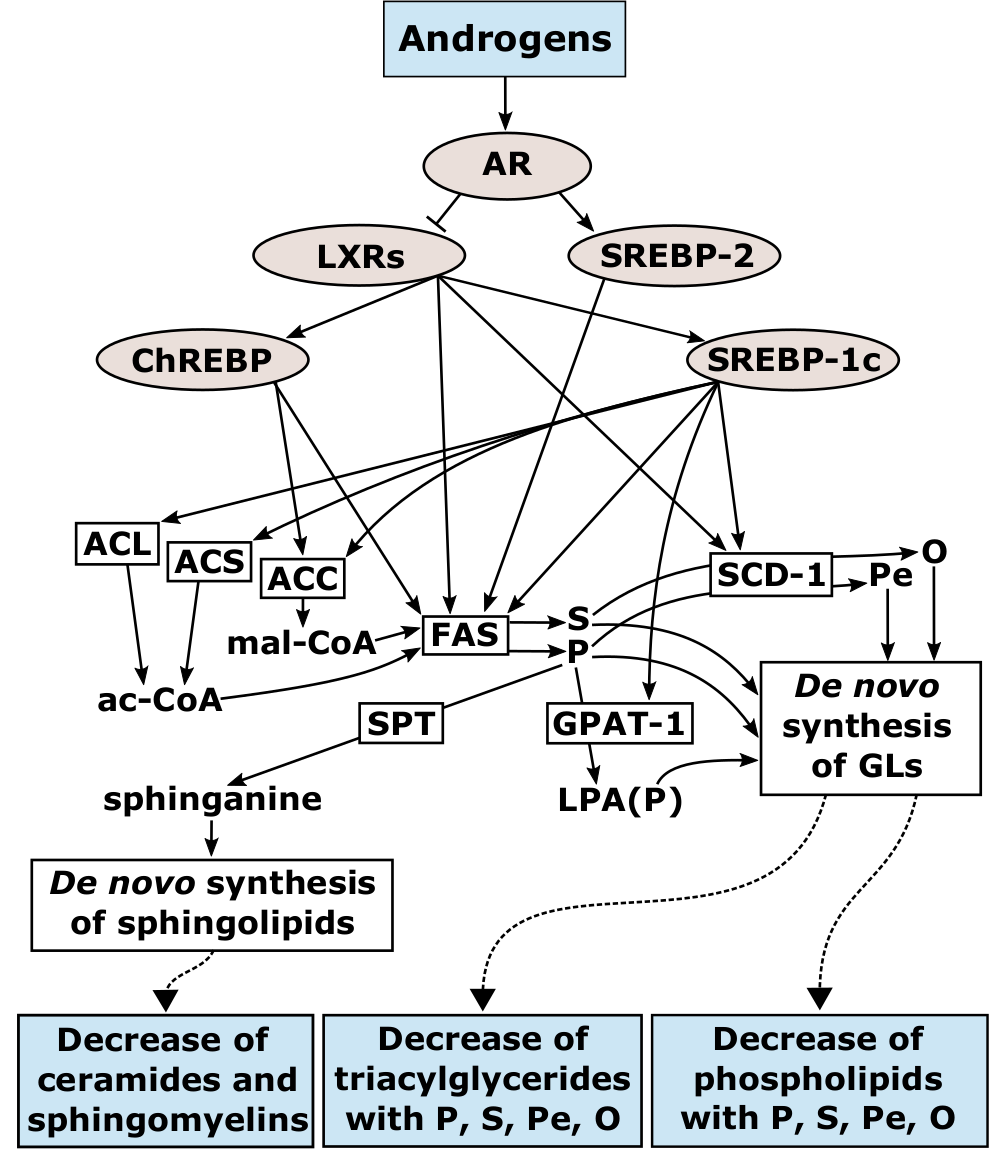


**Figure S.1. Relationship between androgens and lipogenesis according to enzyme regulation and selectivity in the LXR pathway in the light of previous research**

**References**

Choi, S. H., & Ginsberg, H. N. (2011). Increased very low density lipoprotein (VLDL) secretion, hepatic steatosis, and insulin resistance. *Trends in endocrinology and metabolism*, *22*(9), 353–363. https://doi.org/10.1016/j.tem.2011.04.007

Cowart, L. A., & Hannun, Y. A. (2007). Selective substrate supply in the regulation of yeast de novo sphingolipid synthesis. *The Journal of Biological Chemistry*, *282*(16), 12330–12340. https://doi.org/10.1074/jbc.M700685200

Harayama, T., & Riezman, H. (2018). Understanding the diversity of membrane lipid composition. *Nature Reviews. Molecular Cell Biology*, *19*(5), 281–296. https://doi.org/10.1038/nrm.2017.138

Krycer, J. R., & Brown, A. J. (2011). Cross-talk between the androgen receptor and the liver X receptor: implications for cholesterol homeostasis. *The Journal of Biological Chemistry*, *286*(23), 20637–20647. https://doi.org/10.1074/jbc.M111.227082

Mathews, C. K., & Mathews-Van Holde-Appling-Anthony-Cahill (Eds.). (2013). *Biochemistry* (4. ed.). Toronto: Pearson.

Mauvais-Jarvis, F. (2011). Estrogen and androgen receptors: regulators of fuel homeostasis and emerging targets for diabetes and obesity. *Trends in endocrinology and metabolism*, *22*(1), 24–33. https://doi.org/10.1016/j.tem.2010.10.002

Mauvoisin, D., & Mounier, C. (2011). Hormonal and nutritional regulation of SCD1 gene expression. *Biochimie*, *93*(1), 78–86. https://doi.org/10.1016/j.biochi.2010.08.001

Monroy, G., Rola, F. H., & Pullman, M. E. (1972). A substrate- and position-specific acylation of sn-glycerol 3-phosphate by rat liver mitochondria. *The Journal of Biological Chemistry*, *247*(21), 6884–6894.

Smith, S., Witkowski, A., & Joshi, A. K. (2003). Structural and functional organization of the animal fatty acid synthase. *Progress in Lipid Research*, *42*(4), 289–317. https://doi.org/10.1016/S0163-7827(02)00067-X

Tijburg, L. B., Geelen, M. J., & van Golde, L. M. (1989). Regulation of the biosynthesis of triacylglycerol, phosphatidylcholine and phosphatidylethanolamine in the liver. *Biochimica Et Biophysica Acta*, *1004*(1), 1–19. https://doi.org/10.1016/0005-2760(89)90206-3

Wang, B., & Tontonoz, P. (2018). Liver X receptors in lipid signalling and membrane homeostasis. *Nature Reviews. Endocrinology*, *14*(8), 452–463. https://doi.org/10.1038/s41574-018-0037-x

Yu, J., Loh, K., Song, Z.-Y., Yang, H.-Q., Zhang, Y., & Lin, S. (2018). Update on glycerol-3-phosphate acyltransferases: the roles in the development of insulin resistance. *Nutrition & Diabetes*, *8*(1), 34. https://doi.org/10.1038/s41387-018-0045-x
